# Determinants of Confidence in Overall Knowledge About COVID-19 Among Healthcare Workers in South Africa: Results From an Online Survey

**DOI:** 10.3389/fpubh.2021.614858

**Published:** 2021-04-29

**Authors:** Thabang Manyaapelo, Tholang Mokhele, Sibusiso Sifunda, Philisiwe Ndlovu, Natisha Dukhi, Ronel Sewpaul, Inbarani Naidoo, Sean Jooste, Boikhutso Tlou, Mosa Moshabela, Musawenkosi Mabaso, Khangelani Zuma, Priscilla Reddy

**Affiliations:** ^1^Human and Social Capabilities Research Division, Human Sciences Research Council, Pretoria, South Africa; ^2^eResearch Knowledge Centre, Human Sciences Research Council, Pretoria, South Africa; ^3^Human and Social Capabilities Research Division, Human Sciences Research Council, Cape Town, South Africa; ^4^Human and Social Capabilities Research Division, Human Sciences Research Council, Durban, South Africa; ^5^School of Nursing and Public Health Medicine, University of KwaZulu-Natal, Durban, South Africa

**Keywords:** knowledge, confidence, COVID-19, health care workers, South Africa, pandemic

## Abstract

**Background:** Adequate information and knowledge about COVID-19 has been shown to induce the confidence and positive performance among healthcare workers (HCWs). Therefore, assessing the relationship between confidence in knowledge and associated factors among HCWs is vital in the fight against COVID-19. This paper investigates factors associated with HCWs' confidence in their overall knowledge about COVID-19 in South Africa in the early stages of the epidemic.

**Methods:** Data utilized in this paper were from an online survey conducted among HCWs using a structured questionnaire on a data free online platform. The study population were all the medical fraternity in South Africa including medical and nurse practitioners as well as other healthcare professionals. Bivariate and multivariate logistic regression models were performed to examine the factors associated with confidence in HCWs' overall knowledge about COVID-19.

**Results:** Overall, just below half (47.4%) of respondents indicated that they had confidence in their overall knowledge about COVID-19. Increased odds of having confidence in the knowledge about COVID-19 were significantly associated with being male [aOR = 1.31 95% CI (1.03–1.65), *p* < 0.05], having a doctorate degree [aOR = 2.01 (1.23–3.28), *p* < 0.05], being satisfied with the information about COVID-19 guidelines [aOR = 6.01 (4.89–7.39), *p* < 0.001], having received training in 6–8 areas [aOR = 2.54 (1.89–3.43), *p* < 0.001] and having received training in 9–11 areas [aOR = 5.33 (3.81–7.47), *p* < 0.001], and having already treated COVID-19 patients [aOR = 1.43 (1.08–1.90), *p* < 0.001]. Those who were highly concerned with the levels of training of HCWs [aOR = 0.47 (0.24–0.92), *p* < 0.05] had decreased odds of having confidence in their overall knowledge about COVID-19.

**Conclusion:** This study sheds light on the importance of capacitating HCWs with knowledge and adequate relevant training as part of infection prevention control measures during pandemics. Future training and information sharing should be sensitive to knowledge gaps by age, gender, qualifications, professional categories, and experience.

## Introduction

Severe Acute Respiratory Syndrome Coronavirus 2 or SARS-CoV-2 is the virus responsible for the illness now commonly referred to as “corona virus” disease 2019 (COVID-19). This novel coronavirus was first reported in December 2019 with cases emanating from Wuhan city in the Hubei Province of China (1). The World Health Organization (WHO) declared COVID-19 a public health emergency of international concern at the end of January 2020 and subsequently on March 11th, 2020 it was declared a global pandemic (2). In South Africa, the first case was confirmed on March 5th, 2020 and the country proceeded to declare a state of disaster on March 15th, 2020 (3). Approximately 2 weeks thereafter a nation-wide stay at home total lockdown was declared beginning at midnight on the 26th of March 2020, which was effective for 21 days, and then later extended. Only essential services including food production, distribution and sales of food items; pharmacies and medical facilities were given permission to remain open (4).

COVID-19 transmission occurs through droplets, bioaerosols, fomites (5, 6), it also occurs through symptomatic and asymptomatic infected people (7). The most commonly reported symptoms of mild disease include a fever, dry cough, fatigue, and shortness of breath (8). Most people with such mild symptoms are able to recover fully by isolating and self-management at home. However, those who develop severe symptoms such as severe acute respiratory illness will likely require hospitalization (8). Hospitalizations and deaths were found to be increased in patients with underlying comorbidities such as hypertension, diabetes (9), HIV and TB (10). At the time when this study was conducted there were no vaccines or anti-viral pharmaceuticals developed but currently, there are over 40 vaccines undergoing clinical trials with three reported to have completed Phase III with positive results (11). The South African National Department of Health has prioritized the vaccination of healthcare workers targeting 1,250,000 in their vaccine rollout. Healthcare workers play a pivotal role in strengthening the health system as they assist with the full recovery of hospitalized patients diagnosed as positive COVID-19 cases. The HCWs' overall knowledge and understanding of this novel virus underpins their ability to provide optimum care for sick individuals. This knowledge of COVID-19 and translation into practice is particularly important since HCWs belong to the occupational category most threatened by COVID-19 infection globally (12).

Recent studies on HCWs' knowledge, attitude, and practice about COVID-19 reported generally, sufficiently high knowledge, positive attitudes and good practices across several countries (13–17). In another study from an earlier H1N1 pandemic, knowledge was found to be strongly correlated to practice and attitude scores (18). Interestingly, the same study found that higher knowledge scores and a lower level of education were significant predictors of higher practice scores and lastly found that higher knowledge was also a significant predictor of higher attitude scores. These data demonstrate the importance of having adequate knowledge to be able to deliver optimum patient care. Another study on COVID-19 in Uganda reported that factors associated with higher levels of knowledge were age 40 years and older and the use of news media as an information source on COVID-19 (13). Better knowledge has in certain contexts been shown to increase the uptake of preventive measures (19, 20). It needs to be highlighted that knowledge in itself has been shown to have little effect on the behavioral outcome therefore when investigating an intended behavior it would be more beneficial to examine the underlying beliefs associated with that behavior (21). The three elements of knowledge, attitude and practices are linked and form part of the theoretical model in health promotion which seeks to understand underlying beliefs associated with certain behaviors. The behaviors considered to be relevant for this study are the responsive and adequate care for COVID-19 patients that results in recovery and positive patient outcomes.

Acquisition of adequate knowledge has a positive effect on confidence where higher levels of knowledge on personal protective equipment (PPE) were significantly positively correlated to the HCWs confidence in PPE (22). However, in instances where HCWs were found to have low levels of confidence toward COVID-19 care, their poor confidence was associated with the lack of knowledge on optimal infection-prevention measures (23). Studies have reported that one of the better ways to improve student knowledge, skills and self-confidence is to incorporate simulation exercises in their education (24). Such specialized training was shown to significantly improve self-confidence especially when students were given individual attention by qualified practitioners in their field (25). Feeling supported, being self-confident and feeling competent are some of the factors most essential among nurses to facilitate their ability to make effective clinical decisions (26). However, the relationship between confidence and knowledge is not a straightforward one. Some researchers report that having confidence in one's knowledge does not necessarily result in a positive outcome when faced with the magnitude of effort required for future knowledge investment (27). Fischer and Sliwka (27), found that it is the confidence in learning abilities that is more important and that participants with higher levels of confidence in prior knowledge tend to invest less in future learning. Some studies have reported that those who are deemed experts in their field are likely to show higher confidence in their knowledge compared to those who are not considered experts but most importantly their confidence in knowledge tends to be matched with factual knowledge (28). Therefore, what is crucial to highlight is that confidence in knowledge encompasses more than the ability to retrieve information accurately but rather speaks more to the availability and accessibility of this information from ones memory (29). There is a body of literature reporting on the knowledge, attitudes, and practices of HCWs across varying contexts globally (13–17). However, to our knowledge there are no studies reporting on the confidence in knowledge among HCWs. The aim of this paper is to examine the determinants of the confidence in the overall knowledge of COVID-19 for HCWs in South Africa whilst the epidemic was still in its early stages of its trajectory.

## Methods

### Data

Data utilized in this paper were from an online survey conducted from 11th April to 7th May 2020 using a structured questionnaire. The study population was all HCWs aged 18 years and older from all provinces in South Africa. These included medical and nursing practitioners as well as other types of HCWs. An online survey was utilized instead of the face-to-face method as it was administered during lock down level 5 in South Africa and physical distancing was advised.

A data-free mobile messaging platform was utilized to facilitate participation in the online survey by HCWs. Additionally, the study used a comprehensive communication strategy and communication alerts to publicize the call for participation were widely distributed *via* mainstream media channels, including social media, email, radio and television broadcasters, and on local websites; an extensive network of strategic partners in the South African government, science councils, higher education, HCWs' professional councils, healthcare sector partners, non-profit organizations, the private sector, and faith-based and community organizations. These communication alerts included an invitation with the survey link, which when clicked on, directed potential respondents to the online survey. Both a data-free link and a standard internet link to the survey were distributed.

### Measures

The primary outcome variable was confidence in overall knowledge about COVID-19 based on the question, “Do you feel confident in your overall knowledge about COVID-19?” with response options being 1 = yes, 2 = no, and 3 = unsure. These were categorized into a dichotomized primary outcome with 1 = yes and 0 = no (no and unsure).

Explanatory variables were socio-demographic variables such as sex (male or female), age group in years (18–29, 30–39, 40–49, 50–59, 60, and older), population group according to Statistics South Africa (Stats SA) designations (30) (Black African, Colored, White, Indian/Asian, Other), education level [diploma(s)/occupational certificate(s), bachelor's degree, honors/post-graduate diploma, master's degree, specialist qualification, doctorate], professional category (medical practitioner, nurse practitioner, other healthcare worker), employed in public sector (yes or no), private sector (yes or no), other sector (yes or no), and locality for work (urban formal areas, urban informal areas, rural areas).

Other variables included satisfaction with information about COVID-19, risk perception for contracting COVID-19, concerned with levels of training of HCWs, having received training in managing COVID-19, and having treated COVID-19 patient. To assess satisfaction with information about COVID-19, the following question was considered, “Do you feel satisfied with the information being filtered to you from your workplace structures about guidelines for COVID-19?” was presented to respondents with response options being 1 = yes, 2 = no, and 3 = unsure. These responses were further dichotomized into 1 = yes and 0 = no (no and unsure). Risk perception was assessed using the question “How would you rate your personal risk of contracting COVID-19 in the workplace?” with response options being 1 = extremely high risk, 2 = high risk, 3 = moderate risk, 4 = low risk, and 5 = very low risk. These responses were recoded into three categories namely 1 = low risk (very low risk and low risk), 2 = moderate risk and 3 = high risk (extremely high risk and high risk). Concern among HCWs about their levels of training was explored using the question “What is your level of concern regarding each of the following? Levels of training of HCWs about COVID-19” with response options of 1 = not concerned at all, 2 = moderately concerned, 3 = highly concerned, and 4 = extremely concerned. These were recoded into three categories, 1 = not concerned, 2 = moderately concerned, 3 = highly concerned (highly concerned and extremely concerned). Having received training in managing COVID-19 was assessed using the following question, “Have you received any formal training about the management of COVID-19 (referrals, case definitions, management guidelines, infection control etc.)?” with 11 areas of training being (1) Screening people for COVID-19; (2) Referring individuals for testing; (3) The tests that should be done to make the diagnosis; (4) Case definitions; (5) Protocol for workplace infection control; (6) Isolation procedures for patients; (7) Treatment guidelines; (8) Staff transportation; (9) Patients transportation; (10) Visitor policies; and (11) Declaring patients as recovered, with each having response options of 1 = yes, 2 = no, and 3 = n/a. The responses to these 11 items were used to create a received training areas sum score ranging from 1 to 11, where 1 was assigned to each correct response. These were further recorded into four groups, 1 = score of 2 or less training areas, 2 = 3–5 training areas, 3 = 6–8 training areas, and 4 = 9–11 training areas. Having treated COVID-19 patient was assessed with the question “Have you treated/provided care for a patient diagnosed with COVID-19?” with binary responses of yes or no.

### Statistical Analysis

There was no readily available and compiled central database of the total South African HCWs population by demographic characteristics. Therefore, the sampling frame was estimated using information that was readily available from two databases, namely the Health Professions Council of South Africa (HPCSA), and the South African Nursing Council (SANC).

Evidence from other studies has shown that re-weighted online samples can produce response patterns that are statistically similar to population characteristics. Therefore, the survey data were then benchmarked (weighted) to the distribution of South Africa's estimated HCWs population by age, sex, population group, and province using the estimated sampling frame database. Benchmarking was aimed at correcting for potential bias that may result due to the convenient sampling. Benchmarking ensure that the views and perceptions of overrepresented sectors of the HCWs population are scaled downwards and those of less represented are illuminated. This in turn enabled us to increase generalizability to the national sample of HCWs.

Supplementary Table 1 shows the demographic distribution of the weighted analytic sample and the estimated sample frame. The demographic profile of the analytic sample does not differ substantially from the sample frame estimates. Furthermore, Supplementary Table 2 shows relationship between trainings received and confidence in HCWs' knowledge about COVID-19. In addition, this table shows that those who received training in more areas were significantly more likely to be confident in their knowledge about COVID-19 than those who received training in fewer areas (*p* < 0.0001).

Statistical analysis was performed using Stata version 15.0 (31) using data weighted by the “svy” command.

Descriptive statistics with unweighted frequencies and weighted percentages are presented. Differences in confidence in overall knowledge about COVID-19 across the socio-demographic variables were compared using 95% confidence intervals (CIs) and the Chi-square-test. The association between confidence in overall knowledge about COVID-19 and potential explanatory variables was assessed using bivariate logistic regression models. All statistically significant variables from these bivariate logistic regression models were entered into the final multivariate logistic regression model to examine the factors associated with confidence in overall knowledge about COVID-19. Crude and adjusted odds ratios (aOR) with 95% confidence intervals and a *p* < 0.05 was considered statistically significant.

## Results

### Background Characteristics of the Study Sample

The study sample used for this paper was 5,530 respondents, based on the primary outcome variable of confidence in overall knowledge about COVID-19. After benchmarking, females accounted for 76.7% and Black Africans constituted 58.5% of the sample (Table 1). About 28% were 30–39 years old, 31.4% had a Bachelor's degree, and 36.1% were nurse practitioners. Half of the total sample (50.1%) worked in the public sector, 61.9% worked in urban formal areas, and 28.7% worked in Gauteng province. Majority of the healthcare workers (88.1%) reported not having treated COVID-19, which is to be expected as this was still in the early stages of the pandemic.

**Table 1:** Characteristics of the study sample.

	**Sample**	**%**	**95% CI**
**Total**	**5,530**	**100**	
**Sex**			
Female	3,866	76.7	(75.2–78.2)
Male	1,639	23.3	(21.8–24.8)
**Population group**			
Black African	1,210	58.5	(56.7–60.3)
White	2,788	22.4	(21.3–23.6)
Colored	529	12.4	(11.3–13.6)
Indian/Asian	663	5.6	(5.1–6.1)
Other	340	1.0	(0.9–1.2)
**Age group (years)**			
18–29	905	18.2	(16.7–19.8)
30–39	1,653	28.2	(26.5–29.9)
40–49	1,436	24.2	(22.6–25.9)
50–59	922	17.8	(16.2–19.4)
60+	614	11.7	(10.3–13.2)
**Education level**			
Diploma(s)/occupational certificate(s)	982	23.4	(21.7–25.1)
Bachelor's degree	1,654	31.4	(29.5–33.3)
Honors/post-grad diploma	980	16.3	(14.9–17.7)
Master's degree	826	13.1	(11.8–14.6)
Specialist qualification	867	12.4	(11.2–13.7)
Doctorate	221	3.5	(2.8–4.3)
**Professional category**			
Nurse practitioner	1,254	36.1	(34.1–38.1)
Medical practitioner	2,220	30.5	(28.8–32.3)
Other healthcare worker	2,056	33.4	(31.6–35.3)
**Public sector**			
No	3,400	49.9	(48.0–51.9)
Yes	2,128	50.1	(48.1–52.0)
**Private sector**			
No	3,067	67.5	(65.7–69.2)
Yes	2,461	32.5	(30.8–34.3)
**Other sector**			
No	3,916	71.7	(69.8–73.4)
Yes	1,612	28.3	(26.6–30.2)
**Locality for work**			
Urban formal areas	4,064	61.9	(59.8–63.9)
Urban informal areas	888	22.1	(20.4–23.9)
Rural areas	549	16.0	(14.4–17.8)
**Province for work**			
Eastern Cape	409	9.0	(8.0–10.2)
Free state	192	5.3	(4.3–6.4)
Gauteng	1,806	28.7	(27.0–30.4)
KwaZulu-Natal	1,026	21.2	(19.6–22.9)
Limpopo	137	8.4	(7.0–10.1)
Mpumalanga	153	5.5	(4.5–6.7)
North West	180	5.6	(4.6–6.7)
Northern Cape	86	1.8	(1.4–2.4)
Western Cape	1,541	14.6	(13.6–15.6)
**Satisfied with information about COVID-19**			
No	2,369	50.3	(48.3–52.3)
Yes	3,139	49.7	(47.7–51.7)
**Risk perception for contracting COVID-19**			
Low	839	11.2	(10.1–12.3)
Moderate	1,816	27.6	(25.9–29.3)
High	2,875	61.3	(59.4–63.1)
**Concerned with levels of training of HCWs**			
Not concerned	199	2.8	(2.3–3.5)
Moderately concerned	1,573	21.5	(20.0–23.0)
High concerned	3,676	75.7	(74.1–77.2)
**Treated COVID-19 patient**			
No	4,696	88.1	(86.9–89.2)
Yes	797	11.9	(10.8–13.1)

### Confidence in Overall Knowledge About COVID-19

Table 2 summarizes the reported confidence in overall knowledge about COVID-19 by socio-demographic characteristics and other knowledge related variables. Overall, just below half (47.4%) of respondents indicated were confident in their overall knowledge about COVID-19. More Males (56.9%) reported that they were significantly more confident in their overall knowledge about COVID-19 than females (44.5%). The White sub-population group (61.3%), participants aged 60 years and older (63.8%), those with specialized qualification (57.1%) and doctorate (55.9%), medical practitioners (54.7%), and those who worked in the private health sector (56.1%), in urban formal areas (51.1%), and residing in the Western Cape province (59.2%) were significantly more confident in their overall knowledge about COVID-19 than their counterparts.

**Table 2:** Confidence in overall knowledge about COVID-19 by socio-demographic characteristics and knowledge related variables.

	**Total**	**%**	**95% CI**	***p-*value**
**Total**	**5,530**	**47.4**	**(45.4–49.3)**	
**Sex**				
Female	3,866	44.5	(42.1–46.8)	<0.001
Male	1,639	56.9	(53.4–60.4)	
**Population group**				
Black African	1,210	41.0	(37.9–44.1)	<0.001
White	2,788	61.3	(59.2–63.3)	
Colored	529	50.5	(45.8–55.3)	
Indian/Asian	663	50.4	(46.3–54.4)	
Other	340	53.3	(47.2–59.4)	
**Age group**				
18–29	905	38.8	(34.4–43.5)	<0.001
30–39	1,653	40.8	(37.5–44.1)	
40–49	1,436	49.6	(45.7–53.5)	
50–59	922	52.8	(47.7–57.8)	
60+	614	63.8	(57.2–70.0)	
**Education**				
Diploma(s)/occupational certificate(s)	982	42.6	(38.6–46.7)	<0.001
Bachelor's degree	1,654	46.8	(43.2–50.5)	
Honors/post-grad diploma	980	44.4	(39.8–49.1)	
Master's degree	826	49.5	(43.9–55.1)	
Specialist qualification	867	57.1	(51.8–62.2)	
Doctorate	221	55.9	(44.2–67.0)	
**Professional category**				
Nurse practitioner	1,254	41.7	(38.2–45.4)	<0.001
Medical practitioner	2,220	54.7	(51.4–58.0)	
Other healthcare worker	2,056	46.7	(43.5–50.0)	
**Public sector**				
No	3,400	53.8	(51.1–56.4)	<0.001
Yes	2,128	41.0	(38.2–44.0)	
**Private sector**				
No	3,067	43.2	(40.7–45.7)	<0.001
Yes	2,461	56.1	(53.0–59.1)	
**Other sector**				
No	3,916	46.7	(44.3–49.0)	0.253
Yes	1,612	49.2	(45.5–53.0)	
**Locality for work**				
Urban formal areas	4,064	51.1	(48.7–53.4)	<0.001
Urban informal areas	888	41.8	(37.4–46.3)	
Rural areas	549	40.7	(35.2–46.5)	
**Province for work**				
Eastern Cape	409	39.8	(33.7–46.3)	<0.001
Free State	192	49.5	(39.7–59.4)	
Gauteng	1,806	48.9	(45.6–52.2)	
KwaZulu-Natal	1,026	46.3	(42.0–50.5)	
Limpopo	137	35.4	(26.7–45.2)	
Mpumalanga	153	39.4	(29.8–49.9)	
North West	180	48.1	(38.7–57.6)	
Northern Cape	86	50.0	(36.3–63.6)	
Western Cape	1,541	59.2	(55.8–62.4)	
**Satisfied with information about COVID-19**				
No	2,369	21.1	(19.0–23.4)	<0.001
Yes	3,139	73.6	(71.2–76.0)	
**Risk perception for contracting COVID-19**				
Low	839	59.9	(54.5–65.0)	<0.001
Moderate	1,816	52.6	(49.0–56.1)	
High	2,875	42.7	(40.1–45.4)	
**Concerned with levels of training of HCWs**				
Not concerned	199	78.6	(67.3–86.8)	<0.001
Moderately concerned	1,573	69.9	(66.2–73.4)	
High concerned	3,676	40.1	(37.7–42.3)	
**Treated COVID-19 patient**				
No	4,696	46.1	(44.0–48.3)	<0.001
Yes	797	56.6	(51.3–61.7)	

Respondents who were satisfied with information about COVID-19 guidelines being filtered to them from their workplace structures (73.6%), those with low perceived risk of contracting COVID-19 (59.9%), those who were not concerned with levels of training of HCWs (78.6%), and those who said they had treated COVID-19 patients (56.6%) were significantly more confident in their overall knowledge about COVID-19 than their counterparts.

### Factors Associated With Confidence in Overall Knowledge About COVID-19

Table 3 shows factors associated with HCWs' confidence in their overall knowledge about COVID-19. Male respondents [aOR = 1.31 95% CI (1.03–1.65), *p* < 0.05] were significantly more likely to be confident in their overall knowledge about COVID-19 than their female counterparts. Those with doctorate degrees [aOR = 2.01 (1.23–3.28), *p* < 0.05] were significantly more likely to be confident in their overall knowledge about COVID-19 than those with diplomas/occupational certificates.

**Table 3:** Multivariate logistic regression model of factors associated with confidence in overall knowledge about COVID-19.

	**aOR**	**(95% CI)**	***p-*value**
**Sex**			
Female (Ref)			
Male	1.31	(1.03–1.65)	<0.05
**Population group**			
Black African (Ref)			
White	1.18	(0.92–1.51)	0.2
Colored	0.96	(0.72–1.27)	0.767
Indian/Asian	1.08	(0.82–1.43)	0.589
Other	1.02	(0.70–1.48)	0.923
**Age group (years)**			
18–29 (Ref)			
30–39	0.93	(0.69–1.26)	0.658
40–49	1.14	(0.82–1.58)	0.431
50–59	0.94	(0.65–1.36)	0.756
60+	1.28	(0.82–2.00)	0.271
**Education level**			
Diploma(s)/occupational certificate(s) (Ref)			
Bachelor's degree	1.17	(0.88–1.54)	0.277
Honors/post-grad diploma	0.93	(0.68–1.26)	0.629
Master's degree	1.1	(0.78–1.56)	0.596
Specialist qualification	1.32	(0.92–1.90)	0.135
Doctorate	2.01	(1.23–3.28)	<0.05
**Professional category**			
Nurse practitioner (Ref)			
Medical practitioner	0.85	(0.64–1.14)	0.279
Other healthcare worker	1.28	(0.97–1.69)	0.087
**Public sector**			
No (Ref)			
Yes	0.89	(0.69–1.16)	0.399
**Private sector**			
No (Ref)			
Yes	1.05	(0.82–1.35)	0.694
**Locality for work**			
Urban formal areas (Ref)			
Urban informal areas	1.05	(0.80–1.39)	0.722
Rural areas	1.08	(0.79–1.50)	0.622
**Satisfied with information about COVID-19**			
No (Ref)			
Yes	6.01	(4.89–7.39)	<0.001
**Risk perception for contracting COVID-19**			
Low (Ref)			
Moderate	0.89	(0.65–1.23)	0.488
High	0.82	(0.59–1.13)	0.228
**Concerned with levels of training of HCWs**			
Not concerned (Ref)			
Moderately concerned	0.96	(0.48–1.93)	0.919
Highly concerned	0.47	(0.24–0.92)	<0.05
**Number of areas in which participants received training**			
≤ 2 training areas (Ref)			
3–5 training areas	1.36	(0.99–1.86)	0.057
6–8 training areas	2.54	(1.89–3.43)	<0.001
9–11 training areas	5.33	(3.81–7.47)	<0.001
**Treated COVID-19 patient**			
No (Ref)			
Yes	1.43	(1.08–1.90)	<0.05

*aOR, adjusted Odds Ratio; CI, Confidence Level*.

Respondents who were satisfied with the information about COVID-19 guidelines being filtered to them from their workplace structures [aOR = 6.01 (4.89–7.39), *p* < 0.001] were significantly more likely to be confident in their overall knowledge about COVID-19 than those who were not. Healthcare workers who were highly concerned with their levels of training [aOR = 0.47 (0.24–0.92), *p* < 0.05] were significantly less likely to be confident in their overall knowledge about COVID-19 than those who were not concerned at all.

Those who indicated they received training in 6–8 training areas [aOR = 2.54 (1.89–3.43), *p* < 0.001] and those who received training in 9–11 areas [aOR = 5.33 (3.81–7.47), *p* < 0.001] were significantly more likely to be confident in their overall knowledge about COVID-19 than those who received training in two or fewer training areas. This suggests confidence was boosted among those who had received a wider scope of training than those with limited scope. Those who reported that they had already treated COVID-19 patients [aOR = 1.43 (1.08–1.90), *p* < 0.001] were significantly more likely to be confident in their overall knowledge about COVID-19 than those who had not treated such patients, suggesting that direct hands on experience with patient care and management also boosted confidence.

## Discussion

The COVID-19 pandemic has presented HCWs globally with enormous challenges in their work environments. The management of this infectious disease in a hospital setting is critical to the recovery of patients found to present with severe symptoms as well as patient care on an outpatient basis to manage recovery and prevent progression to severe disease. The present study's objective was to investigate factors associated with healthcare workers' confidence in their overall knowledge about COVID-19 as the epidemic had newly emerged in South Africa. This study found that less than half of the participating HCWs reported having confidence in their overall knowledge about COVID-19 This finding must be viewed in the context of the time frame that the study was done, which was just as cases started to increase in wave 1 of the emergence of the epidemic in South Africa and can be considered to be a baseline of HCWs confidence in these areas.

It is expected that knowledge about this novel virus is time sensitive in the local healthcare setting and it is dependent on several factors including receiving timely information from the global setting as well as the local setting. Previous studies reporting on the H1N1 pandemic, found that overall knowledge about that virus had also been lower during the initial stages of the pandemic in April 2009 but this improved around August 2009 (32). The current study found that those who were aged 60 years and older had higher confidence in their knowledge. This finding together with higher confidence among those who had treated covid cases suggests that the experience contributed to confidence level. Indeed studies have shown that overall self-esteem progressively increases up to age 70 (33). The finding of practical experience of working with the virus bestowing more confidence is comparable to a study in Henan, China where frontline HCWs reported having more confidence in ompared to non-frontline workers (34). The same study found that HCWs with higher levels of knowledge had more confidence in managing COVID-19 patients.

The present study also found that those with college degrees and certificate qualifications were found to have higher level of overall confidence in their knowledge of COVID-19 compared to those without degrees and certificates. Studies have shown that self-confidence has a strong relationship with academic achievement but this strength is partially dependent on the societal context (35).

The present study also found that male HCWs had higher levels of confidence in their overall knowledge of COVID-19 compared to female HCWs Recent studies have reported that society tends to readily accept men as confident but only accepts women as confident if there is an element of prosocial orientation (36). Therefore, the level of confidence in overall knowledge reported by women in this present study could be underestimated. However, this could also be related to the HCW professional category as nurses are predominately female in the South African context. Consistent with observations elsewhere, this study indicated that nurses had a lower confidence in overall knowledge about COVID-19 as compared to medical practitioners (23). The lower confidence in overall knowledge among nurses warrants further attention to identify specific areas of need. However, previous studies have postulated that in the prevention and management of infectious diseases intensive training of all HCWs is essential. In the case of COVID-19, health facility owners/managers are responsible for provision of HCW training (37, 38). The Global Health Security report flagged training gaps among HCWs for epidemics in general. The issues related to inadequate training among nurses is not unique to South Africa. The International Council of Nurses highlighted training gaps among nurses in areas pertaining to formal infection prevention and control (IPC) and PPE for airborne transmitted infections (39). Training programs can improve HCWs' confidence levels so that regardless of academic achievement and level, overall knowledge of COVID-19 can be increased.

Healthcare workers in the Western Cape province had higher levels of confidence in overall knowledge about COVID-19 than HCWs in other provinces. The Western Cape had experienced higher infection and transmission rates compared to other provinces in the country at the initial and progressive stages of the pandemic (40). This could partially explain the higher levels of confidence since these HCWs were dealing with relatively more cases daily compared to their counterparts in other provinces. Furthermore, this study also found that HCWs who had reported treating COVID-19 patients had higher levels of confidence in their knowledge. This further confirms the higher confidence seen from HCWs in the Western Cape.

The study had strengths and some limitations. The strength of the study was the relatively large sample size of HCWs who participated on the study, representing all major professional categories in South Africa. In addition, the strength of this study is that it utilized an online survey methodology which provided real-time results as the COVID-19 pandemic was unfolding during the period when the country was under the highest lockdown level which restricted movement of people and rendered it difficult to reach frontline HCWs through face-to-face contact. A shortcoming of the study is inadequate measure for knowledge of COVID-19 among HCWs. The questionnaire did not have a fully encompassing knowledge about COVID-19 indicator that could be measured in this paper. A previous study (41) had found that overall knowledge among the wider South African population was relatively high (over 90%) therefore it was assumed to be higher among HCWs.

However, the fact that HCWs who received training in more areas on COVID-19 management were more likely to be confident in their knowledge about COVID-19 than those who received training in fewer areas showed that knowledge (acquired through training) leads to confidence in HCWs' overall knowledge about COVID-19. Participation through online platforms could be biased to HCWs who had access to technology and internet as well as the time and motivation to do so. To mitigate the impact of this limitation, the survey data were then benchmarked to the distribution of South Africa's estimated HCWs population by age, sex, population group, and province using the estimated sampling frame database. One of the limitations of the study could have been the fact that the study was conducted during the first wave in South Africa and there is a possibility that the confidence levels among HCWs could have increased after the second wave. Based on the above mention strengths and limitations, the authors believe that the findings from this study contribute to the COVID-19 research with evidence from the developing countries.

## Conclusion

Less than half of the HCWs in South Africa had confidence in their overall knowledge about COVID-19 during the early stages of the pandemic and this is expected to improve over time as the pandemic develops and HCWs receive more formal and informal training, knowledge and experience. This study sheds light on the importance of capacitating HCWs with knowledge and adequate training as part of infection prevention control measures for epidemic preparedness. Intensified future training activities for HCWs should be inclusive and sensitive to knowledge gaps by age, gender, qualifications, professional categories, and levels of experience.

## Data Availability Statement

The raw data supporting the conclusions of this article will be made available by the authors, without undue reservation.

## Ethics Statement

The studies involving human participants were reviewed and approved by Human Sciences Research Council Research Ethics Committee (REC) Protocol Number: REC 5/3/20. The patients/participants provided their written informed consent to participate in this study.

## Author Contributions

PR conceived the study. TMa, TMo, SS, and PN conceptualized the paper. TMo conducted the data analysis. TMa and TMo drafted the manuscript. ND, RS, IN, SJ, BT, MMo, MMa, KZ, and PR contributed to designing the analyses, interpreting results, and made substantial contributions to the manuscript. All authors read and approved the final manuscript before submission.

## Conflict of Interest

The authors declare that the research was conducted in the absence of any commercial or financial relationships that could be construed as a potential conflict of interest.
