# Supplementary material for: Determinants of Confidence in Overall Knowledge About COVID-19 Among Healthcare Workers in South Africa: Results From an Online Survey
Source: Front Public Health. 2021 Apr 29;9:614858. doi: 10.3389/fpubh.2021.614858 (PMC8118123; doi:10.3389/fpubh.2021.614858)
Supplement: Supplementary file 1 [file Table_1.DOCX]

**Supplementary Table 1: Comparison of weighted vs unweighted vs sampling frame**

|  | **Estimated sampling frame** | | | | **Weighted analytic sample** | |
| --- | --- | --- | --- | --- | --- | --- |
|  | **Health professionals^1^ (HPCSA)** | **Nurses^2^ (SANC)** | **Total Health care workers** | |  |  |
|  | Number | Number | % | Number | % | Number |
| Total | 172 640 | 285 704 | 100.0 | 458 344 | 100.0 | 5 530 |
| Province |  |  |  |  |  |  |
| Eastern Cape | 12 259 | 29 533 | 9.1 | 41 792 | 9.0 | 409 |
| Free State | 10 119 | 13 398 | 5.1 | 23 517 | 5.3 | 192 |
| Gauteng | 59 955 | 74 044 | 29.2 | 133 999 | 28.7 | 1 806 |
| KwaZulu-Natal | 27 170 | 71 030 | 21.4 | 98 200 | 21.2 | 1 026 |
| Limpopo | 11 671 | 28 839 | 8.8 | 40 510 | 8.4 | 137 |
| Mpumalanga | 9 709 | 14 878 | 5.4 | 24 587 | 5.5 | 153 |
| North West | 7 682 | 18 002 | 5.6 | 25 684 | 5.6 | 180 |
| Northern Cape | 3 561 | 3 747 | 1.6 | 7 308 | 1.8 | 86 |
| Western Cape | 30 513 | 32 233 | 13.7 | 62 746 | 14.6 | 1 541 |
|  |  |  |  |  |  |  |
| Gender |  |  |  |  |  |  |
| Male | 71 920 | 28 162 | 21.8 | 100 082 | 23.3 | 1 639 |
| Female | 100 720 | 257 542 | 78.2 | 358 262 | 76.7 | 3 866 |
|  |  |  |  |  |  |  |
| Race |  |  |  |  |  |  |
| African | 82 584 | 203 993 | 62.5 | 286 577 | 58.5 | 1 210 |
| White | 58 922 | 32 285 | 19.9 | 91 207 | 22.4 | 2 788 |
| Coloured | 11 854 | 40 570 | 11.4 | 52 424 | 12.4 | 529 |
| Indian | 16 246 | 7 428 | 5.2 | 23 674 | 5.6 | 663 |
| Other | 3 034 | 1 429 | 1.0 | 4 463 | 1 | 340 |
|  |  |  |  |  |  |  |
| Age^3^ |  |  |  |  |  |  |
| 18–29 years | 73 545 | 21 900 | 20.8 | 95 445 | 18.2 | 905 |
| 30-39 years | 50 066 | 79 772 | 28.3 | 129 838 | 28.2 | 1 653 |
| 40–49 years | 25 551 | 80 696 | 23.2 | 106 247 | 24.2 | 1 436 |
| 50-59 years | 13 639 | 64 952 | 17.1 | 78 591 | 17.8 | 922 |
| >=60 years | 9 840 | 38 384 | 10.5 | 48 224 | 11.7 | 614 |

1. Source: Health Professional Association of South Africa (HPCSA)
2. Source: South African Nursing Council (SANC)
3. The estimated proportions of medical practitioners by age and nurses by population group were based on Shisana et al. (2002). O Shisana, E Hall, KR Maluleke et al. (HSRC, MEDUNSA & MRC). The Impact of HIV/AIDS on the Health Sector. National survey of health personnel, ambulatory and hospitalised patients and health facilities 2002.
