# Supplementary material for: Determinants of Confidence in Overall Knowledge About COVID-19 Among Healthcare Workers in South Africa: Results From an Online Survey
Source: Front Public Health. 2021 Apr 29;9:614858. doi: 10.3389/fpubh.2021.614858 (PMC8118123; doi:10.3389/fpubh.2021.614858)
Supplement: Supplementary file 2 [file Table_2.DOCX]

**Supplementary Table 2: Training received in individual topic areas and number of topic areas in which participants received training by confidence in knowledge about COVID-19**

|  | **Confidence in knowledge about COVID-19** | | | | | | |
| --- | --- | --- | --- | --- | --- | --- | --- |
|  | **Total** | **Yes** | | **No** | | **p value** |  |
| **Training received in individual topic areas:** |  | **%** | **95% CI** | **%** | **95% CI** |  |  |
| Screening people for COVID-19 | 4,026 | 53.9 | [51.5-56.2] | 46.1 | [43.8-48.5] | <0.0001 |  |
| Referring individuals for testing | 3,693 | 58.9 | [56.4-61.3] | 41.1 | [38.7-43.6] | <0.0001 |  |
| The tests that should be done to make the diagnosis | 3,542 | 58.6 | [56.0-61.1] | 41.4 | [38.9-44.0] | <0.0001 |  |
| Case definitions | 3,407 | 59.2 | [56.6-61.7] | 40.8 | [38.3-43.4] | <0.0001 |  |
| Protocol for workplace infection control | 4,331 | 55.9 | [53.6-58.2] | 44.1 | [41.8-46.4] | <0.0001 |  |
| Isolation procedures for patients | 3,796 | 57.1 | [54.7-59.5] | 42.9 | [40.5-45.3] | <0.0001 |  |
| Treatment guidelines | 3,181 | 63.3 | [60.6-65.9] | 36.7 | [34.1-39.4] | <0.0001 |  |
| Staff transportation | 1,610 | 70.0 | [66.4-73.4] | 30.0 | [26.6-33.6] | <0.0001 |  |
| Patient transportation | 1,869 | 65.0 | [61.6-68.2] | 35.0 | [31.8-38.4] | <0.0001 |  |
| Visitor policies | 3,780 | 55.1 | [52.6-57.5] | 44.9 | [42.5-47.4] | <0.0001 |  |
| Declaring patients as recovered | 1,480 | 69.2 | [65.2-73.1] | 30.8 | [26.9-34.8] | <0.0001 |  |
| **Number of topic areas in which participants received training** |  |  |  |  |  |  |  |
| ≤ 2 training areas | 940 | 23.1 | [19.5-27.0] | 77 | [73.0-80.5] |  |  |
| 3-5 training areas | 1157 | 28.8 | [25.1-32.7] | 71.2 | [67.3-74.9] |  |  |
| 6-8 training areas | 1729 | 52.1 | [48.5-55.7] | 47.9 | [44.3-51.5] |  |  |
| 9-11 training areas | 1700 | 76.8 | [73.4-79.9] | 23.2 | [20.1-26.6] | <0.0001 |  |
